# A draft genome of a field-collected *Steinernema feltiae* strain NW

**DOI:** 10.21307/jofnem-2020-003

**Published:** 2020-03-17

**Authors:** Zhen Fu, Yuxiang Li, Axel A. Elling, William E. Snyder

**Affiliations:** 1 Department of Entomology, Washington State University, Pullman, WA; 2 Current: Department of Entomology, Texas A&M University, College Station, TX; 3 Department of Plant Pathology, Washington State University, Pullman, WA; 4 Current: Bayer Crop Science, Cary, NC; 5 Current: Department of Entomology, University of Georgia, Athens, GA

**Keywords:** Entomopathogenic nematode, Biological control, Genome, *Steinernema feltiae*

## Abstract

Advances in sequencing technologies have accelerated our understanding of the complex genetic network of organisms and genomic divergences that are linked to evolutionary processes. While many model organisms and laboratory strains have been sequenced, wild populations are underrepresented in the growing list of sequenced genomes. Here, we present a *de novo* assembly of *Steinernema feltiae*, strain NW, collected from a working agricultural field in south central Washington, USA. Leveraging Pacific Biosciences (PacBio) long reads, we sequenced strain NW to a high depth (99×). The resulting *de novo* assembly is significantly larger than the previous assembly generated from the laboratory strain SN, with a noticeable improvement in continuity and completeness. Comparative analysis of two assemblies revealed numerous single nucleotide polymorphisms (SNPs), breakpoints, and indels present between the two genomes. This alternative genome resource and annotation could benefit the research community to examine the genetic foundation of evolutionary processes as well as genomic variation among conspecific populations.

The genome encodes the entire inheritance messages of living organisms, serving as the foundation for biological, cellular, and molecular processes that are essential for development and reproduction. Knowledge of genomes advances the understanding of complex gene networks and assists in the engineering of crops and biological control agents for trait improvement (Bolger et al., 2014; Lu et al., 2016; Rodríguez-Leal et al., 2017). Research efforts to date have largely focused on model species and laboratory strains. Recently, however, the rapid progress of high-throughput sequencing and computational algorithms have begun to enable sequencing of field-collected species and strains (Wang et al., 2018; Wu et al., 2018; Kingan et al., 2019), which could be divergent from their laboratory-maintained congeners due to the lack of artificial selections (Palková, 2004; Barriere and Felix, 2005). Scrutinizing these wild populations and species might shed light on genetic changes during both domestication processes and natural selection, paving the way for better biodiversity conservation, trait selection, and targeted breeding (Fuentes-Pardo and Ruzzante, 2017; Wang et al., 2018; Wu et al., 2018).

The entomopathogenic nematode *Steinernema feltiae* is widely applied to control insect pests, such as scarab larvae, fungus gnats, and lepidopteran larvae (Toba et al., 1983; Jess and Bingham, 2004; Lacey et al., 2006; Lacey and Georgis, 2012). As an obligate parasite, *S. feltiae* relies on the toxin produced by its symbiotic bacterium *Xenorhabdus bovienii* to kill insect hosts, which the nematodes then consume. The tripartite of bacterium–nematode–insect presents an interesting model to examine the mutualistic relationship between nematodes and symbiotic bacteria, as well as insect immune responses (Goodrich-Blair, 2007; Castillo et al., 2011). Dillman et al. (2015) published the first draft genome of *S. feltiae*, offering a comprehensive resource for understanding the evolution of parasitism genes. The strain SN used by Dillman et al. (2015) has long been maintained under laboratory conditions. Under such conditions, organisms could undergo significant changes in genome structure, gene copy numbers as well as accumulating mutations that are associated with adaptation to artificial conditions (Farslow et al., 2015; Sterken et al., 2015).

Here, we report a genome assembly and annotation for *S. felitae* NW a naturally occurring entomopathogenic nematode strain, which was shown to be effective in causing mortality of insect hosts (unpubl. data). Entomopathogens that coevolve with their local insect hosts could exhibit higher efficacy on killing insect pests than commercial strains (Noosidum et al., 2010). Thus, a genomic resource from a field-collected entomopathogenic strain could offer valuable insights into local adaptation as well as trait improvement for this broadly applied biopesticide.

## Materials and methods

### Nematode strain

*S. feltiae* strain NW was collected using trap insect technique with modification (see Kaya and Stock, 1997) from a potato field near Patterson, WA under organic management. Specifically, a trap consisted of 10 wax moth, *Galleria mellonella*, larvae (Nature’s Way, Ross, OH) in a sealed mesh bag was buried 15 cm deep in the soil, and the trap was retrieved 48 hr later. Nematodes were extracted from dead larvae using White traps (White, 1927). A week later, infective juveniles (IJs) were collected and kept at 12°C. To reduce heterozygosity, 20 IJs were used to establish an inbred colony. These IJs were used to infect a single wax moth, and seven days after infection, IJs were recovered using a White trap (White, 1927). Approximately 200 emerged IJs were used to infect 10 wax moths for two more times. Emerged IJs were washed three times in sterilized tap water and stored in −80°C until DNA purification.

### DNA purification

Approximately 10,000 IJs were thawed and frozen twice. High molecular weight genomic DNA was extracted using a phenol chloroform protocol described in the study of Donn et al. (2008). The DNA pellet was re-suspended in 100 μl 10 mM Tris-Cl (pH 8.5) buffer. Washington State University’s Genomic Core Facility (Pullman, WA) performed library preparation and sequencing. Three batches of sequencing with different chemistries were conducted: XL-XL, P4-C2, and P5-C3. In total, 42 SMRT cells (24 cells of XL chemistry, 7 cells of P4-C2 chemistry, and 11 cells of P5-C3 chemistry) were included in the sequencing.

### Read filtering and genome assembly

We used bash5tool.py algorithm in the SMRT analysis pipeline (v2.3) to extract subreads from all 42 SMRT cells with minReadScore set to 0.75. Subreads were used for genome assembly. We tested two genome assembly programs initially: Canu (v1.3; Koren et al., 2017) and Celera (v8.3rc2; Myers et al., 2000). For both assemblers, we started with the default setting. Canu outperformed Celera in running time and memory use. Canu also generated an assembly with fewer contigs and greater N50. Thus, we picked Canu for further fine-tuning to improve assembly quality. For the final assembly, we used sprai (v0.9.9.18, http://zombie.cb.k.u-tokyo.ac.jp/sprai) to correct errors for the subreads, and we imported corrected reads into Canu to perform *de novo* assembly.

### Quality control

Once we obtained an assembly with the best continuity and lowest number of contigs, we removed contigs that had low read support (<20 reads). The read coverage information was indicated in Canu (v1.3; Koren et al., 2017). Often, contigs with low read support were highly fragmented and not informative in building gene models during the annotation process. Next, we polished the assembly by first aligning raw PacBio-h5 files to the assembly using the program pbalign following consensus calling using Quiver (SMRT analysis pipeline (v2.3)). Finally, we queried each contig in the assembly to genome sequences of the endosymbiont *X. bovienii* (GenBank assembly accession: GCA_000027225.1) using BLASTN (Altschul et al., 1990). Contigs showed homology to any sequence of *X. bovienii* with bit score >200 were removed in the assembly.

### Genome alignment

MUMmer (v4.09; Marçais et al., 2018) was used to align our assembly to the genome assembly of SN. Program dnadiff in MUMmer was used to summarize the comparative analysis of the two genomes. Program nucmer provided a 1-to-1 and all-vs-all comparisons of the two genomes, and this analysis was limited to the 100 longest contigs/scaffolds to minimize noise.

### Genome annotation and evaluation

Because the genome assembly of strain NW seemed to be divergent from the previously published genome of strain SN, we annotated this genome to present as an alternative resource for the research community. To do this, we modeled and identified potential repeat region of the *S. feltiae* NW genome based on a repeat library kindly provided by WormBase using RepeatModeler (v1.0.11, http://www.repeatmasker.org/RepeatModeler/). After that, we used RepeatMasker (v4.0.9; Smit et al., 2013-2015) to soft mask the genome. Two RNA-seq data sets (BioProjects accession numbers PRJNA488495 and PRJNA283145, Chang et al., 2019), were aligned to the NW genome assembly using program HISAT2 (v2.1.0; Kim et al., 2015) with the default setting. The resulting bam files from HISAT2 were used as RNA-seq evidence to train gene models in BRAKER2 (Hoff et al., 2015). The generated annotation file in GFF3 format from BRAKER2, along with the protein FASTA file of strain SN (Dillman et al., 2015), were incorporated into the program MAKER (v2.31.10; Cantarel et al., 2008) to synthesize gene models. BUSCO (v3.02; Waterhouse et al., 2017) was used to evaluate the completeness of the genome based on a set of universal single-copy orthologs across Nematoda.

### Data archiving

The whole genome assembly was archived in NCBI with WGS accession number MQUG00000000. PacBio subreads were deposited in SRA under BioProject PRJNA353610. Genome in GFF3/GTF format along with predicted mRNA, CDs, and protein sequences were deposited in WormBase ParaSite and will be available from release 15 and forward.

## Results

### PacBio sequencing of strain NW

In total, 7,166,944 reads (read length ranging from 500 bp to 39,605 bp, median=1,429 bp, mean=1,681 bp) were generated from 42 SMRT cells across three chemistries. Reads generated in our study were sufficient to cover the genome 145-fold, assuming the genome size of 82 Mb as previously described (Dillman et al., 2015). Such high coverage would adequately overcome the high error rate often associated with long reads generated from PacBio platforms (Rhoads and Au, 2015).

### *De novo* assembly

The final assembly used in the downstream analysis was generated in Canu (Koren et al., 2017) with an error rate=0.03, and raw reads covered the genome assembly 99-fold. After removing contigs that were likely contaminated with *X*. *bovienii*, and contigs with low coverage support (<20 reads), the final assembly contained 4,876 contigs with N50 (contigs) of 60,433 bp. The assembled genome size of NW is significantly larger (47%) than SN with noticeable improvement regarding the number of contigs and continuity (N50, Table 1). In addition, our assembly of NW contained no ‘gaps’ (i.e., unknown sequences between two contigs of which order and orientation are known; Clum, 2018) in comparison to numerous gaps totaling 2,769,616 bp in the SN genome; this would facilitate annotation of the genome (e.g., Dallery et al., 2017).

**Table 1.** Summary of the genome assembly of strain NW in comparison to strain SN.

	Strain NW	Strain SN
*Assembly*		
Total bases (bp)	121,603,260	82,627,153
No. of scaffolds	na	5,839
Scaffold N50	na	47,851
No. of contigs	4,678	59,024
Contig N50	60,433	3,650
No. of gaps (bp)	0	2,769,616
Longest scaffolds (bp)	1,315,981	1,446,875
No. of predicted genes	32,304	36,434
Total no. of amino acids	12,632,601	12,195,137
*BUSCO assessment*		
Complete BUSCOs	87.27%	84.32%
Complete single copy	76.68%	80.55%
Complete duplicated copy	10.59%	3.77%
Fragmented	7.33%	8.15%
Missing	5.40%	7.54%
*Alignment*		
Aligned Seqs	4,279 (91.47%)	5,418 (92.79%)
Aligned Bases	92,999,444 (76.48%)	74,444,081 (90.10%)
1-to-1	49,949	49,949
Total length (1-to-1)	72,468,269	72,538,432
Avg. identity (1-to-1)	97.07	97.07
M-to-M	133,667	133,667
Total length (M-to-M)	106,859,791	106,997,101
Avg. identity (M-to-M)	95.69	95.69
*Genomic variation*		
Breakpoints	261,902	252,282
Relocations	8,456	5,231
Translocations	13,595	16,494
Inversions	1,411	1,952
Insertions	105,325	74,670
Total SNPs	907,706	907,706

### Comparative genomic analysis

**Figure 1:**
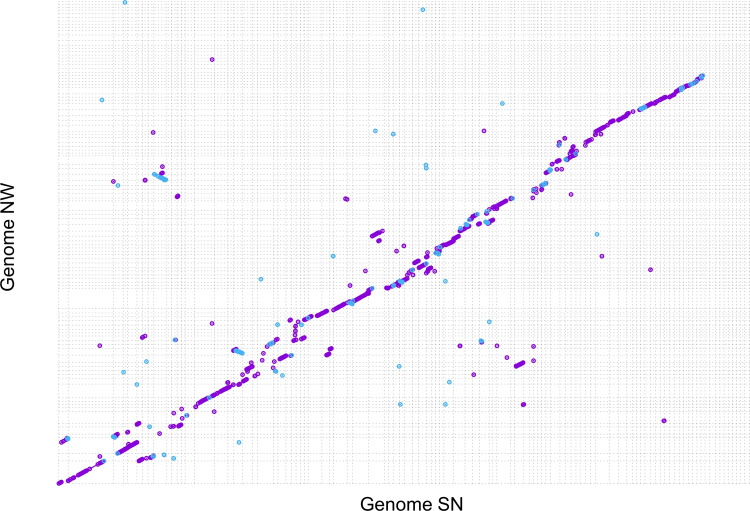
Genome alignment of strains SN and NW. Only top 100 longest scaffolds from SN (laid across the x-axis) and top 100 longest contigs from NW (y-axis) were shown here to minimize noise. Each contig/scaffold is shown between two lines (vertical for SN and horizontal for NW) along the axes. A colored dot is plotted wherever the two sequences agree; the forward matches are shown in purple, while the reverse matches are shown in blue. If the two genomes were perfectly identical, a series of purple dots would be drawn diagonally.

We performed a comparative genomic analysis of our NW assembly with the earlier SN assembly using a whole genome alignment approach. Over 90% of the contigs/scaffolds from the two genomes aligned, totaling 94 Mb and 74 Mb from the NW and SN assemblies, respectively. In total, 24 percent (29 Mb) of the NW sequences failed to align to the SN assembly, indicating divergence between the two strains. Genome alignment determined that there were 49,949 1-to-1 best alignments between these two assemblies with an average identity of 97%, totaling 72.5 Mb of the sequences from both genomes. There were 133,667 many-to-many alignments with a similar average identity of 96%. There were 907,706 SNPs detected in common between the two genomes in addition to numerous indels, breakpoints, and allocations (Table 1). The majority of the top 100 longest contigs/scaffolds from the two assemblies aligned with the presence of insertions and rearrangements. We also noted that a few contigs/scaffolds failed to align (absence of dots at the top right of Figure 1). Despite our improvement of the assembly, neither of these two genomes has assembled to the chromosome level.

### Genome annotation

Because our genome seemed to be quite divergent from the assembly of strain SN with many structural variations and SNPs, we proceeded to annotate the genome with evidence generated from RNA-Seq alignment. Despite the larger assembled genome size, our annotation predicted rather fewer (12% fewer genes) number of genes/proteins compared to the previous annotation of SN genome (Table 1). However, the total length of amino acids in our annotation is longer than SN, suggesting a longer or greater number of full-length genes. Furthermore, our NW genome had a slightly higher percentage of completeness compared to the SN genome (Table 1).

## Discussion

We sequenced and annotated the genome of the field-collected strain of *S. feltiae* NW, a broadly applied biological control agent for insect pests (Toba et al., 1983; Jess and Bingham, 2004; Lacey et al., 2006; Lacey and Georgis, 2012). A previous genome project required multiple mate libraries (Dillman et al., 2015), in comparison to the single library generated in our study, suggesting that a simplified procedure could generate high quality genome assembly. The work presented here improves the current genome resources for this species in a few ways, including improving the continuity and the completeness of the draft genome. Furthermore, this resource represents a draft genome of a naturally occurring strain that was collected from a working agricultural field, which could be phylogenetically divergent from its laboratory counterpart that was sequenced previously (Palková, 2004; Barriere and Felix, 2005). The genome size of the NW assembly is significantly larger than the assembly of SN provided by Dillman et al., (2015), reaching 121.6 Mb. Genome size variation within a species is not uncommon (Bosco et al., 2007; Huang et al., 2013). Many factors including differing environmental conditions, unrecognized speciation events, and artifacts of the techniques used for sequencing could all contribute to apparent intraspecific variation in genome size (Biemont, 2008; Cires et al., 2010; Gunn et al., 2015; Leinaas et al., 2016). Thus, a survey of multiple *S. feltiae* strains using flow cytometry could provide a more accurate estimation of genome size, or instead verify the apparent differences in genome size seen across the two genome sequencing efforts completed thus far.

Though in-depth comparative genomic analyses are beyond the scope of this work, we suggest that further comparative genome analyses of field versus laboratory conspecific strains would shed light on the genomic variations across different populations. Neither the SN or NW genomes have chromosome-level scaffolds, as only two contigs of the NW assembly and only one scaffold of the SN assembly are over 1 Mb in length. A different approach to sequencing and library preparation, such as long-range linkage, could be used to generate a chromosome-level scaffold with multiple genome assemblies as foundations (Kolmogorov et al., 2018). With the advancement of sequencing technologies, the cost of sequencing a genome will further decline. In a single run, with sequencing technologies a higher output of data might be generated, and deeper coverage achieved, to resolve uncertainties for the *S. feltiae* genomes that are currently available. We expect that research groups could utilize the genome resources provided here to expand our understanding of genome networks, evolutionary processes, and genomic variation of field-collected nematode populations.
